# A web-based, patient driven registry for Angelman syndrome: the global Angelman syndrome registry

**DOI:** 10.1186/s13023-017-0686-1

**Published:** 2017-08-01

**Authors:** Kathryn R. Napier, Megan Tones, Chloe Simons, Helen Heussler, Adam A. Hunter, Meagan Cross, Matthew I. Bellgard

**Affiliations:** 1 Centre for Comparative Genomics, Murdoch University, Perth, WA 6150 Australia; 2 Mater Research, Centre for Children’s Health Research, South Brisbane, QLD 4101 Australia; 3 Foundation for Angelman Syndrome Therapeutics Australia, Salisbury, QLD 4107 Australia; 4 Mater Research, University of Queensland, Children’s Health Queensland Hospital and Health Service, Brisbane, QLD 4101 Australia

**Keywords:** Angelman syndrome, Disease registry, Global, Interoperable, Open source, Patient reported, Rare disease, Registry framework

## Abstract

Angelman syndrome (AS) is a rare neurodevelopmental disorder that is characterised by severe global developmental delays, ataxia, loss of speech, epilepsy, sleep disorders, and a happy disposition. There is currently no cure for AS, though several pharmaceutical companies are anticipating drug trials for new therapies to treat AS. The Foundation for Angelman Therapeutics (FAST) Australia therefore identified a need for a global AS patient registry to identify patients for recruitment for clinical trials.

The Global AS Registry was deployed in September 2016 utilising the Rare Disease Registry Framework, an open-source tool that enables the efficient creation and management of patient registries. The Global AS Registry is web-based and allows parents and guardians worldwide to register, provide informed consent, and enter data on individuals with AS. 286 patients have registered in the first 8 months since deployment.

We demonstrate the successful deployment of the first patient-driven global registry for AS. The data generated from the Global AS Registry will be crucial in identifying patients suitable for clinical trials and in informing research that will identify treatments for AS, and ultimately improve the lives of individuals and their families living with AS.

## Background

Angelman syndrome (AS) is a rare neurodevelopmental disorder caused by the loss of function of UBE3A, an imprinted, maternally expressed gene on chromosome 15 [1, 2]. The syndrome was first described by Dr. Harry Angelman in 1965 [3], and is characterised by severe global developmental delays, ataxia, loss of speech, epilepsy, sleep disorders, and a happy disposition [4–6]. The developmental delay of AS is generally noticed by six to 12 months of age, with the clinical diagnosis usually made between 1 and 4 years of age [7].

The estimated prevalence of AS is between 1:15,000–24,000 [8, 9], and there is currently no cure. Individuals with AS have a normal lifespan, and normally receive medical therapy for seizures and physical, communication, and behavioural therapies to help improve their quality of life. Current research is investigating the molecular mechanisms by which UBE3A deficiency results in AS, and possible gene therapy treatments. With several pharmaceutical companies anticipating drug trials for new therapies to treat AS, the Foundation for Angelman Therapeutics (FAST) Australia identified a need for a global AS patient registry to further understand the syndrome and to identify patients for recruitment for clinical trials.

Patient registries are crucial tools used to collate information about rare diseases such as AS from affected individuals around the world. Registries serve a number of important purposes, including the identification of individuals suitable for clinical trials and the collection of data in a uniform manner that can inform research. We have developed a Rare Disease Registry Framework (RDRF) to enable the efficient deployment of web-based registries [10–12].

The RDRF is an open-source tool that empowers registry administrators to construct web-based patient registries with minimal software developer effort. The RDRF incorporates a modular design with a graphical user interface (GUI) that allows users to dynamically create all data elements (DEs) that define a patient registry, which can then be shared across registries. The RDRF takes a conceptual approach to the design and development of patient registries to ensure access, security, privacy, and sustainability [10, 13–15], and has been used to deploy national and international registries [16–19].

We describe the deployment of the Global AS Registry utilising the RDRF, through engagement and collaboration with FAST Australia board members, researchers and clinicians from Mater Research (Brisbane) and the Royal Children’s Hospital (Melbourne), and researchers and software developers from the Centre for Comparative Genomics at Murdoch University. The Global AS Registry is a worldwide initiative that will collect data on individuals with AS through a series of online surveys in order to build the largest and most comprehensive global collection of information on AS to date. It is the first online patient-driven registry for AS, with parents and caregivers of individuals with AS driving the collection of data.

The Global AS Registry will help medical professionals and researchers to learn more about individuals with AS, and will create new opportunities to gain insight and understanding about AS. The Global AS Registry will act as an important tool for both facilitating research and enabling clinical trial sponsors to quickly identify suitable patients for studies.

### Registry governance

The Global AS Registry is governed by the Global AS Registry Governance Board, and the Data Curator is responsible for the management of registry activities.

**Fig. 1:**
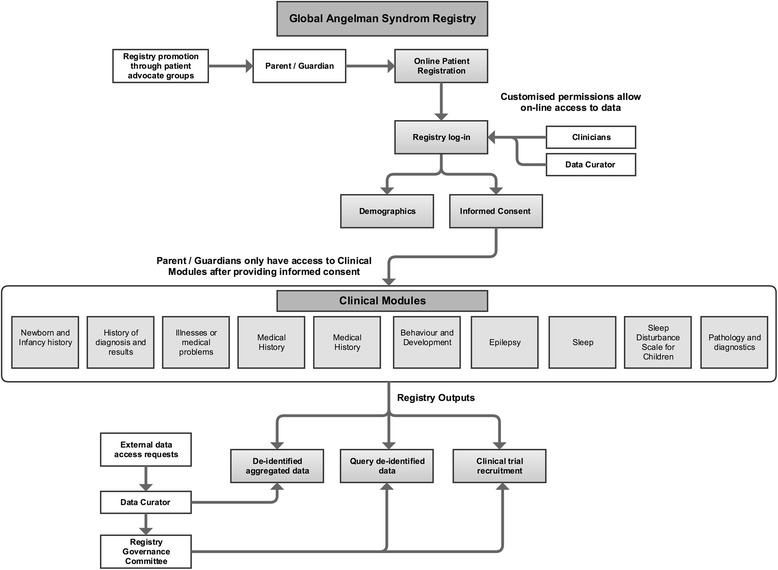
Functions of the Global Angelman Syndrome Registry. The formatting of this figure is adapted from Napier et al. [18]

All requests for data are received by the Data Curator and, depending on the type of requests, are reviewed by the Governance Board (Fig. 1). The Global AS Registry is patient-reported, with parents or guardians of individuals with AS registering their details, providing informed consent to participate in the registry, and completing a series of online questionnaires organised into specific Modules. Due to the ability of the RDRF to assign permissions to multiple levels of users, parent and guardian users can only view the patients they have added to the registry, and only the Data Curator has administration privileges and access to data from all patients defined in the registry. The Mater Health Services Human Research Ethics Committee (HREC/13/MHS/76), Mater Human Research Governance Committee (RG-16-078-AM01) and Murdoch University Human Research Ethics Committee (2016/216) reviewed and approved this study.

### Registry deployment, system architecture, and security

The Global AS Registry is web-based and accessed online [20]. The registry was created using the RDRF, and is hosted and maintained by the Centre for Comparative Genomics at Murdoch University in Western Australia. The RDRF is open-source, with the source code [21] and the project page [22] available online.

The RDRF is built on top of Django 1.8 [23] utilising PostgreSQL [24], HTML, CSS, YAML [25], Javascript, jQeury [26] and Bootstrap [27]. The RDRF is typically deployed via Docker containers [28] using uWSGI [29] and nginx [30]. The Django secure package middleware settings are enabled by default in the RDRF, including secure socket layer (SSL) security (encrypts all web traffic to and from the application), cross-site request forgery (CSRF) checking, and login restrictions of all views. Additionally, identifying demographic information is stored separately to any clinical data (see Napier et al. [18]).

### Patient registration, informed consent, and adding patients

Parents and guardians of individuals with AS register through an online registration page [31]. Select demographic details on the parent/guardian users and ‘patients’ (individuals with AS) are captured, with automatic account verification emails sent to each user upon registration. After the parent/guardian user has verified their account, they are prompted to provide informed consent through the Consent Module prior to clinical Modules being made available to them (Fig. 1).

Upon logging into the registry, parent/guardian users may easily add additional patients. All patients are linked to a parent/guardian user, who provides informed consent and completes each Module on their behalf.

### Modules

Parent/guardian users are guided through a series of online questionnaires organised into Modules, including: Newborn and infancy history, History of diagnosis and results, Illnesses or medical problems, Medical history, Behaviour and development, Epilepsy, Medications and interventions, Sleep, Sleep Disturbance Scale for Children, and Pathology and diagnostics. Longitudinal data collection will occur for selected modules approximately once a year. The RDRF enables the collection of longitudinal data in a variety of contexts, through both time-stamping of data entry in a data field and time stamping of data according to an ‘assessment’ or ‘form’ date [16]. The baseline Minimum Data Set is described in Tones et al. (Megan Tones, Meagan Cross, Chloe Simons, Kathryn R Napier, Adam A Hunter, Matthew I Bellgard, Helen Heussler: Research Protocol: The Initiation, Design and Establishment of the Global Angelman Syndrome Registry, submitted). 

## Results and discussion

The Global AS Registry was deployed in September 2016, with 286 registrations received in the first 8 months since deployment from 32 countries. Approximately half of all patients have provided informed consent, with 35% of patients commencing or completing clinical data entry through the progression through the numerous Modules. Initial statistics on reported key diagnostic measures are described in Tones et al. ﻿(Megan Tones, Meagan Cross, Chloe Simons, Kathryn R Napier, Adam A Hunter, Matthew I Bellgard, Helen Heussler: Research Protocol: The Initiation, Design and Establishment of the Global Angelman Syndrome Registry, submitted)﻿.

The deployment of the Global AS Registry is the result of extensive stakeholder engagement with researchers, clinicians, patient advocacy groups, families, and software developers, driven by FAST Australia. The continued success of the Global AS Registry will be due to the passion of the Angelman Syndrome community to contribute data to the registry, the continued promotion and management of the registry through FAST Australia and the Data Curator and Governance Committee, and the flexibility and sustainability of the RDRF.

Through its modular design, the RDRF allows the Global AS Registry to continue to evolve over time. For example, new Modules and DEs are easily added to the registry as required, and consent questions can be amended, added or removed. Because the RDRF allows the dynamic creation and management of all aspects of registry creation (DEs, sections, Modules, consents, and management of user permissions) by users with little or no software development experience, the registry can be easily managed and updated by the Data Curator. Due to its open-source nature, future developments, enhancements and feature requests are automatically included in the registry, with the Global AS Registry benefiting from design input from other registries (and vice versa).

Future enhancements to the Global AS Registry will include features such as internationalisation, which will expand the availability of the registry to non-English speaking participants. Future improvements will link clinician log-in and view of their patients to the optional consent to contact clinicians provided by parents/guardians, which will enable the clinical verification of patient-reported data. The RDRF also allows longitudinal data to be easily captured and visualised, which may be incorporated into the Global AS Registry at a future date.

## Conclusions

We have demonstrated the successful deployment of the first patient-driven global registry for Angelman Syndrome utilising the RDRF. The Global AS Registry is web-based, which allows parents and guardians of individuals with AS from around the world to register and contribute patient data to the registry in a secure manner. The data generated from the Global AS Registry will be crucial in identifying patients suitable for clinical trials and in informing research that will identify treatments for AS, and ultimately improve the lives of individuals and their families living with AS.

## Abbreviations

AS: Angelman syndrome
CSRF: Cross-site request forgery
DEs: Data elements
FAST: Foundation for Angelman Syndrome Therapeutics
GUI: Graphical user interface
RDRF: Rare Disease Registry Framework
SSL: Secure socket layer
